# visPIG - A Web Tool for Producing Multi-Region, Multi-Track, Multi-Scale Plots of Genetic Data

**DOI:** 10.1371/journal.pone.0107497

**Published:** 2014-09-10

**Authors:** Matthew Scales, Roland Jäger, Gabriele Migliorini, Richard S. Houlston, Marc Y. R. Henrion

**Affiliations:** 1 Division of Genetics and Epidemiology, The Institute of Cancer Research, Surrey, United Kingdom; 2 Department of Mathematics, Imperial College London, London, United Kingdom; Emory University School Of Medicine, United States of America

## Abstract

We present VISual Plotting Interface for Genetics (visPIG; http://vispig.icr.ac.uk), a web application to produce multi-track, multi-scale, multi-region plots of genetic data. visPIG has been designed to allow users not well versed with mathematical software packages and/or programming languages such as R [1], Matlab®, Python, etc., to integrate data from multiple sources for interpretation and to easily create publication-ready figures. While web tools such as the UCSC Genome Browser [2] or the WashU Epigenome Browser [3] allow custom data uploads, such tools are primarily designed for data exploration. This is also true for the desktop-run Integrative Genomics Viewer (IGV) [4],[5]. Other locally run data visualisation software such as Circos [6] require significant computer skills of the user. The visPIG web application is a menu-based interface that allows users to upload custom data tracks and set track-specific parameters. Figures can be downloaded as PDF or PNG files. For sensitive data, the underlying R [1] code can also be downloaded and run locally. visPIG is multi-track: it can display many different data types (e.g association, functional annotation, intensity, interaction, heat map data,…). It also allows annotation of genes and other custom features in the plotted region(s). Data tracks can be plotted individually or on a single figure. visPIG is multi-region: it supports plotting multiple regions, be they kilo- or megabases apart or even on different chromosomes. Finally, visPIG is multi-scale: a sub-region of particular interest can be 'zoomed' in. We describe the various features of visPIG and illustrate its utility with examples. visPIG is freely available through http://vispig.icr.ac.uk under a GNU General Public License (GPLv3).

## Introduction

In genetic research there is an increasing need to integrate multiple datasets, sometimes quite different in nature, for analysis or visualisation. For example, in genome-wide association studies (GWAS) association p-values are commonly annotated with evolutionary conservation data, RNAseq data, ChIP-seq data or functional information, such as ChromHMM [7] output. Often such datasets are on vastly different scales; for example a researcher might fine-map a particular association signal, often involving a single linkage-disequilibrum (LD) block of no more than a few hundred to a few thousand base-pairs, and then use C-based techniques such as 4C [8],[9], Hi-C [10] or ChIA-PET [11] to identify potential long-range interactions spanning tens of kilobases, megabases or several chromosomes.

Presenting all these datasets within a single, easy-to-interpret figure is challenging. Furthermore, it is generally not possible to plot each of the different datasets using the same software. In practice, many researchers produce separate plots, some generated by web resources such as the UCSC Genome Browser or the Broad Institute's SNAP web application [12], which are then combined using image editing software such as Adobe® Photoshop® or Microsoft PowerPoint. This is not ideal as it is error-prone when multiple tracks are aligned.

To address these deficiencies we have developed the Visual Plotting Interface for Genetics, visPIG – http://vispig.icr.ac.uk which allows users to produce figures containing multiple (epi-)genetic data tracks, with options to plot multiple regions and zoom in on specific sub-regions. One key advantage of using visPIG to produce such multi-track, multi-scale and multi-region figures is that the tracks are aligned correctly down to a single base-pair.

## Results and Discussion

visPIG has been developed to have three core capabilities: it can display multiple data tracks across multiple regions at multiple scales. Importantly, visPIG has been designed to be easy to use; specifically, users can access it via a web interface, selecting the files to be uploaded and adjusting plotting parameters through menu panels. Furthermore, at inception it was required that visPIG produces publication-grade graphs that need no subsequent image processing before inclusion in a scientific article. Interactivity, as required for data exploration, was of secondary importance.

**Figure 1:**
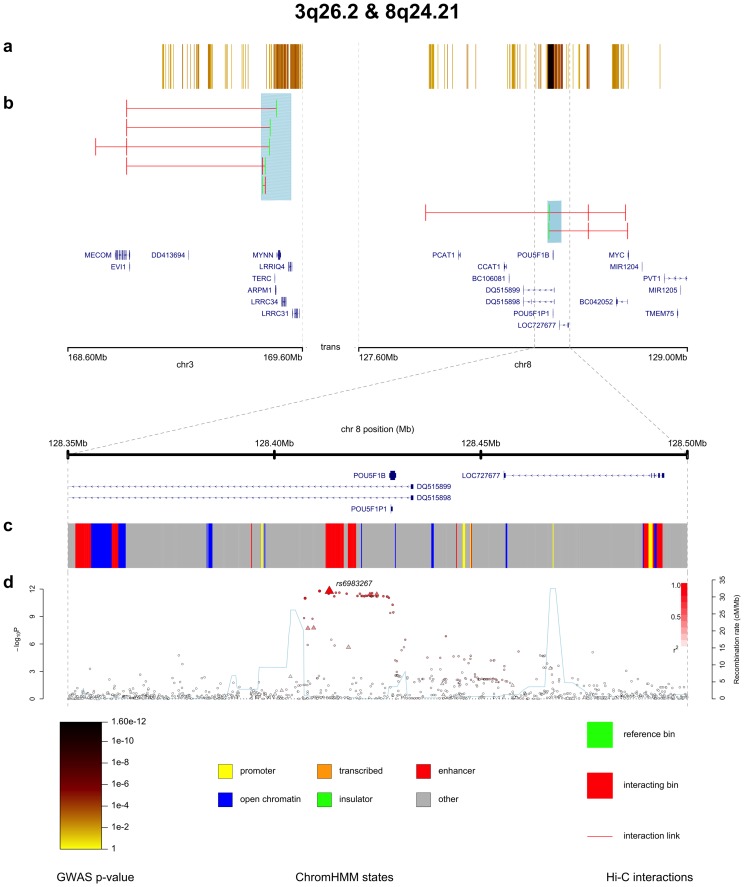
Annotated CRC GWAS p-values for 3q26.2 and 8q24.21, using data from [14] and [15]. The data tracks plotted across the two regions, 3q26.2 and 8q24.21, are **a.** association trend test p-values from a CRC GWAS as a colour intensity track, **b.** significant Hi-C interactions (3 kb resolution; determined from Hi-C experiments on 3 CRC cell-lines, LS174T, LoVo and Colo205), **c.** ChromHMM functional annotation, **d.** CRC GWAS trend test association p-values as a SNAP plot (with SNP type (imputed/typed), 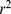 values and recombination rate). The figure includes a title, gene tracks for both scales and a legend.

**Figure 2:**
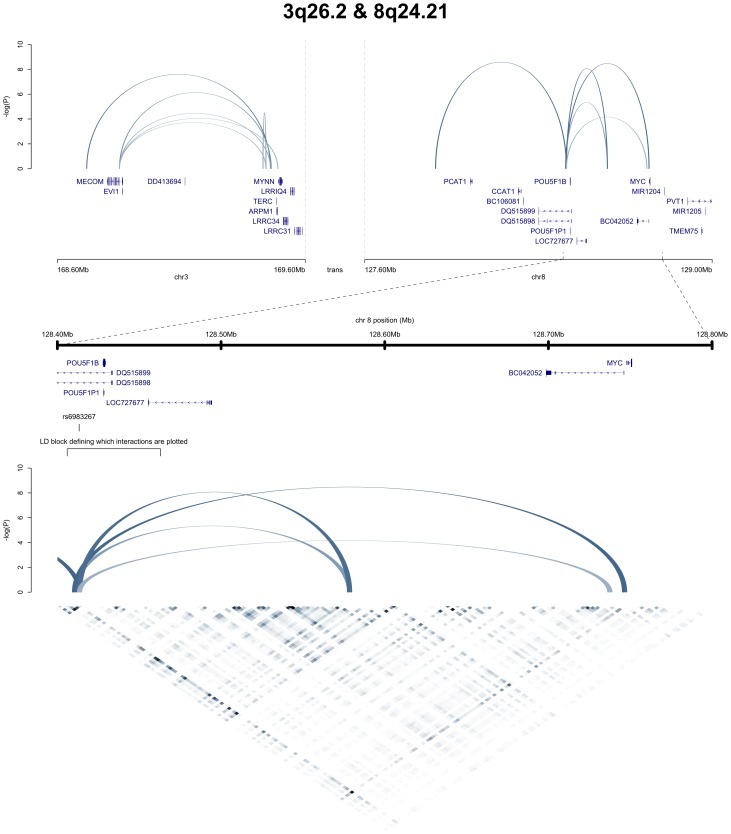
Hi-C interactomes of 3q26.2 and 8q24.21, using data from [15]. This figure shows the same two regions as Figure 1, 3q26.2 and 8q24.21. The data tracks plotted are the significant 3 kb Hi-C interactions (determined from 3 CRC cell-lines, LS174T, LoVo, Colo205), displayed as arches this time, as well as the corresponding, un-normalised Hi-C reads from the LS174T cell-line as a heat map. Also shown are some manual annotations to highlight the LD block within which one end of the significant interactions have to lie to be plotted, as well as the top CRC risk associated SNP in the LD block, rs6983267.

Figures 1 and 2, which have been generated using only visPIG without additional image processing, showcase the utility of the software.

Figure 1 shows association p-values from a colorectal cancer (CRC) GWAS across two regions (3q26.2 and 8q24.21), annotated with Hi-C and ChromHMMM data. The figure demonstrates all three main features of visPIG: i) ability to plot two distinct regions from two different chromosomes, ii) display of multiple data types and iii) zooming in on one specific sub-region. Furthermore, the software allows a title, a legend and track letters for easily referencing the individual data tracks in the caption. Figures 1a 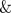 1b show how visPIG allows direct comparison of the GWAS p-values and the 3kb Hi-C interactomes of 3q26.2 and 8q24.21. Note that the two regions are shown on exactly the same scale. Zooming in on the region directly surrounding the highest CRC association signal, rs6983267, makes it possible to study the association data in more detail for the LD block containing rs6983267 (Figure 1d), and to highlight the functional features in this region (Figure 1c).

All input files and parameter values necessary to produce Figure 1 can be downloaded from the example section of the visPIG website.

Figure 2 shows the same two regions as in Figure 1, but displays only Hi-C data. The interactions are plotted in a different style and visPIG's heatmap and feature annotation tracks are showcased. Plotting Hi-C interactions as arches, allows to display the strength of individual interactions; here the p-values of the interactions determine the height and colour intensity of the arches. Zooming in on a sub-region shows the raw, binned Hi-C data that has been used to determine which interactions are significant.

There are a number of visualisation programs and toolkits available. These include the WashU Epigenome Browser [3], the Integrative Genomics Viewer (IGV) [4],[5], the UCSC Genome Browser [2], Circos [6] and the Broad Institute's SNAP regional association plotting interface [12]. Table 1 lists the capabilities of these applications. While some applications, notably IGV, the WashU Epigenome Browsers and the UCSC Genome Browser, are better suited for data exploration, visPIG is the only application that can produce publication-ready graphs with the above mentioned capabilities. Circos has similar capabilities (multi-track, -region 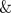 -scale), but has no interface, making it less accessible to users not familiar with executing programs from a terminal, legends have to be added manually and all figures produced by Circos are circular, which may not be appropriate for all situations. The WashU Epigenome Browser is an extremeley powerful data exploration and visualisation tool and also has similar capabilities than visPIG. It is multi-track and multiple panels can be juxtaposed, which allows plotting several regions at different scales. In visPIG's case the different regions are always plotted at exactly the same scale, with only zoomed regions being at a different scale. The Epigenome Browser is more flexible, but in practice it is difficult to get different regions to be plotted at exactly the same scale, and we were unable to indicate where the zoomed panel is located on the full view. The panels will also share the same tracks, whereas for a zoomed view one might want to highlight different data. Legends are available, but are not included on the output figure, onto which they need to be added manually.

**Table 1:** Comparison of visPIG with other genetic data visualisation tools.

application	multi-track	multi-region	multi-scale	type 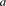	interactive	format 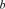	legend 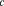	graphics 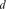
visPIG	√	√	√	w,c	√ 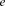	pdf, png	√	v,r
WashU Epigenome Browser	√	√ 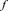	√ 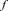	w	√	svg		v
IGV	√		√ 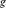	w,g	√	png		r
UCSC Genome Browser	√		√ 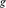	w	√	pdf, png		v,r
Circos	√	√	√	c		svg, png		v,r
SNAP plot				w, c		pdf, png	√	v,r

The comparison has been restricted to the capabilities of visPIG. Some of the other tools have additional features that are not listed here.

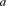
: w = web application, g = locally run graphical user interface, c = locally run from a terminal command line

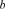
: figure output format

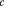
: ability to add a legend onto the output figure

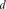
: v = vector graphics, r = raster graphics

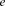
: possible for some features (e.g. all graphical parameters, the type and order of tracks displayed, the zoom view), but not fully interactive; e.g. to change the plotted regions, the region file needs to be edited and re-uploaded

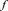
: multiple panels can be juxtaposed, which can be used to either show multiple regions and/or zoom in on a specific sub-region; difficult to get different regions on exactly identical scales; we were unable to indicate the location of the zoomed panel on the full view

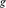
: multi-scale in the sense that one can interactively zoom in on a region, but never more than one scale displayed at a given time.

We conclude that apart from directly programming one's own toolkit using R, Matlab®, Python or similar, we are not aware of any other software that combines visPIG's three core capabilities (plotting multiple datasets for multiple regions at multiple scales), can add add legends directly onto the output figure and has an easy-to-use graphical user interface.

## Materials and Methods

The main visPIG code has been written in R. The complete R code can be downloaded from the visPIG website and does not require installation of any additional R libraries. The R code is intended to be run locally from the command line (i.e. without any graphical user interface as provided on the webpage). Running visPIG locally is required for users with sensitive data that cannot be uploaded to the visPIG server, or with very large data files. The R code is provided freely under a GPLv3 license.

The web application is generated by the R library shiny [13], and hosted on a linux server running the server software shiny-server, which has been developed for hosting R shiny applications. The menu layout has been designed using html and css.
